# The Patient Outcomes Research To Advance Learning (PORTAL) Network Adult Overweight and Obesity Cohort: Development and Description

**DOI:** 10.2196/resprot.5589

**Published:** 2016-06-15

**Authors:** Deborah R Young, Beth A Waitzfelder, David Arterburn, Gregory A Nichols, Assiamira Ferrara, Corinna Koebnick, Ayae Yamamoto, Matthew F Daley, Nancy E Sherwood, Michael A Horberg, Lee Cromwell, Kristina H Lewis

**Affiliations:** ^1^Kaiser Permanente Southern CaliforniaDepartment of Research & EvaluationPasadena, CAUnited States; ^2^Kaiser Permanente HawaiiCenter for Health ResearchHonolulu, HIUnited States; ^3^Group Health Research InstituteSeattle, WAUnited States; ^4^Kaiser Permanente NorthwestCenter for Health ResearchPortland, ORUnited States; ^5^Kaiser Permanente Northern CaliforniaDivision of ResearchOakland, CAUnited States; ^6^Kaiser Permanente ColoradoInstitute for Health ResearchDenver, COUnited States; ^7^HealthPartners InstituteBloomington, MNUnited States; ^8^Mid-Atlantic Permanente Research InstituteRockville, MDUnited States; ^9^Kaiser Permanente GeorgiaAtlanta, GAUnited States; ^10^Wake Forest Baptist Medical CenterDepartment of Epidemiology & PreventionWinston-Salem, NCUnited States

**Keywords:** overweight, obesity, race and ethnic diversity, pre-diabetes, diabetes

## Abstract

**Background:**

The Patient-Centered Outcomes Research Institute (PCORI) created a new national network infrastructure to enable large-scale observational comparative effectiveness research across diverse clinical care settings. As part of testing the feasibility of this effort, each clinical data research network (CDRN) was required to construct cohorts of patients, including one of patients with overweight and obesity.

**Objective:**

The aim of this paper is to report on the development of the Patient Outcomes Research to Advance Learning (PORTAL) overweight and obese cohort, which includes patients from 10 health plans located across the United States.

**Methods:**

Information was gathered from each plan’s electronic health records (EHR). Eligibility included 18 years of age or older, a valid height and weight in 2012 or 2013, and body mass index (BMI) greater than 22.9 kg/m^2^. Pre-diabetes and diabetes status was defined using the American Diabetes Association (ADA) criteria, using lab values of glycated hemoglobin (HbA1c) or fasting glucose available in the EHR. Hypertension was identified from the International Classification of Diseases (ICD) diagnosis codes. Individuals were classified into BMI categories: healthy weight (23.0-24.9 kg/m^2^), overweight (25.0-29.9 kg/m^2^), obese class 1 (30.0-34.9 kg/m^2^), obese class 2 (35.0-39.9 kg/m^2^), obese class 3 (40.0-49.0 kg/m^2^), and obese class 4 (>50.0 kg/m^2^).

**Results:**

A cohort of 5,293,458 non-pregnant adults was created. Weight status was 20.39% (1,079,289/5,293,458) healthy weight, 40.40% (2,138,520/5,293,458) overweight, 22.78% (1,205,866/5,293,458) obese class 1, 9.86% (521,872/5,293,458) obese class 2, 5.59% (295,786/5,293,458) obese class 3, and 0.98% (52,125/5,293,458) obese class 4. Race/ethnicity was 49.02% (2,594,776/5,293,458) non-Hispanic white, 22.89% (1,211,677/5,293,458) Hispanic, 10.40% (550,608/5,293,458) Asian, 10.83% (573,506/5,293,458) black, and 6.59% (348,830/5,293,458) other. About 34.33% (1,817,438/5,293,458) met the definition of hypertension, 20.49% (1,660,940/5,293,458) of individuals met the criteria for pre-diabetes, and 14.98% (793,069/5,293,458) met criteria for diabetes. Prevalence of pre-diabetes and diabetes varied across health plans to a greater extent than expected based on hypertension prevalence and BMI status variability.

**Conclusions:**

This large, race, ethnic, and geographically diverse cohort will be useful for future studies of rare exposures or outcomes and differences in health care practices.

## Introduction

In 2014, the Patient-Centered Outcomes Research Institute (PCORI) funded 11 Clinical Data Research Networks (CDRN) and 18 Patient-Powered Research Networks to develop a National Patient-Centered Clinical Research Network (PCORnet), with the purpose of building a common infrastructure across the CDRNs to enable highly representative future clinical outcomes research. The goal of PCORnet is to "transform clinical research by engaging patients, care providers, and health systems in collaborative partnerships to improve healthcare and advance medical knowledge." One of the CDRNs is the Patient Outcomes Research to Advance Learning (PORTAL) network. PORTAL combines four health care delivery systems that have about 11 million members enrolled across nine states (CA, CO, GA, HI, MD, MN, OR, VA, WA) and the District of Columbia, reaching into most regions in the United States and offering a diverse patient population.

The PORTAL health care systems are previously described [1]. In brief, PORTAL includes all Kaiser Permanente regions (Hawaii, Northwest [Northern Oregon and Southwest Washington], Northern California, Southern California, Colorado, Mid-Atlantic States [Maryland, Virginia, and District of Columbia], and Georgia [through 2015]), Group Health Cooperative (Washington), HealthPartners (Minnesota and Wisconsin), and Denver Health. Individuals of all the health care systems except for Denver Health are insured (public or private); Denver Health is a safety net institution that provides medical services regardless of ability to pay.

All CDRNs were required to develop three cohorts to demonstrate each network’s ability to identify individuals with a condition of interest and to test the commonality of data elements across sites. They also were required to field a survey of the cohorts to test the ability to reach out to patients. One of the pre-specified cohorts common to all of the PCORnet CDRNs was a cohort of individuals with obesity. The PORTAL overweight and obesity cohort was defined as adult members of our health care systems during 2012 or 2013 that were overweight or obese, defined as having a body mass index (BMI) greater than or equal to 23.0 kg/m^2^. Although overweight is defined as BMI greater than 25 kg/m^2^we recognize that the World Health Organization (WHO) recommends lower overweight and obesity cut points for Asians: 23.0-27.4 kg/m^2^for overweight and greater or equal to 27.5 kg/m^2^for obesity [2]. Given that our health plans have a significant number of Asian individuals, we chose this lower cut point so future studies can examine health risks for Asians deemed overweight by WHO recommendations.

We constructed a cross-sectional cohort of adults enrolled in any of the PORTAL health plans; all of those meeting eligibility criteria are considered cohort members. For all sites except Denver Health, we first identified health plan members with at least 12 months of continuous membership between January 1, 2012 and December 31, 2013, and who were at least 18 years of age on December 31, 2013. Members were further restricted to those who had a weight recorded during 2012 or 2013, had a height recorded in the electronic health record (EHR), and who were not pregnant during 2012-2013. For Denver Health, the initial eligibility criteria included all adults who had a primary care encounter during 2012 or 2013 because Denver Health, as a safety-net organization, does not enroll members.

## Methods

### Data Harmonization

Each health care system has its unique methods of capturing its electronic health care data, resulting in information that widely varies in terms of content, format, and structure, thus requiring consistent data standards and terminology. We used the Health Care Systems Research Network (formerly HMO Research Network) Virtual Data Warehouse (VDW) for data extraction. The VDW is a federated database in which all data reside at each health system behind each site’s secure system, or firewall [3]. The data model consists of taking the clinical and claims datasets from the individual health care systems and converting them into a series of identical dataset standards, automated processes, and common data dictionaries. This allows for a single Statistical Analysis System (SAS) program to be written and distributed to other sites with a minimum of site-specific customization. Sites typically return the datasets to the lead site within 2 weeks. Future studies using data from the PORTAL cohort will use the PCORnet common data model (CDM), which is the data structure built for all PCORnet networks. The CDM and VDW have similar data structures; sites run a program that extract data from the VDW into the CDM. The PCORnet CDM was being developed concurrently with the PORTAL cohort; thus, we used the VDW for data extraction.

Kaiser Permanente Southern California (KPSC) is the lead site for the cohort and obtained its institutional review board’s (IRB) approval for human subjects protections for the research. The IRBs at the other sites reviewed the protocol and subsequently ceded review to the KPSC IRB.

### Weight and Height

Weight is routinely measured as part of obtaining vital signs during outpatient clinic visits. Height is typically assessed less often, as it is considered to be more static. If BMI was not available in the EHR, it was calculated. If more than one weight, height, or BMI was in the EHR in 2012-2013, the most recent value was used. EHR records of heights less than 4 ft or equal to or greater than 8 ft, and weights less than 50 lbs or equal or greater than 1000 lbs were considered implausible and were removed from the data set. Similarly, calculated BMI less than 5 kg/m^2^or equal to or greater than 90 kg/m^2^were excluded. A total of 6954 (0.11%, 6954/6,255,688) individuals were excluded from the cohort because they had no biologically plausible weight, height, or BMI values.

We categorized individuals as healthy weight (BMI 23.0-24.9 kg/m^2^), overweight (25.0-29.9 kg/m^2^), obese class 1 (30.0-34.9 kg/m^2^), obese class 2 (35.0-39.9 kg/m^2^), obese class 3 (40.0-49.9 kg/m^2^), or obese class 4 (>50 kg/m^2^) [4]. We classified Asian/Pacific Islanders in the same manner for this initial analysis.

### Race and Ethnicity

Race and ethnicity was obtained from self-report during enrollment into the health plan, during a health care encounter, or from birth certificates (if applicable). Individuals had the option to identify themselves as Asian, Black or African American, Hispanic, Native Hawaiian or other Pacific Islander, American Indian or Alaskan Native, White, or other. If the information was not available in the VDW or individuals identified themselves as belonging to another race or ethnic group, the individual was categorized as "other/unknown."

### Education and Income

Our health plans do not routinely collect individual-level data on educational attainment or income levels, so investigators rely on neighborhood-level information to estimate socioeconomic status. Neighborhood education and income were estimated using geospatial entity object codes (geocodes) that linked addresses to 2010 US census data at the block group level. The probability of different education levels within a block group was used to calculate individual averages. The probability of different family and household income levels within a block group was used to calculate individual averages.

### Pre-Diabetes and Diabetes

Pre-diabetes was defined by the American Diabetes Association (ADA) and from the work of Schmittdiel et al as follows: if during the study period the EHR had (1) at least one HbA1C between 5.7% and 6.4%, or (2) at least one fasting plasma glucose measurement between 100 and 125 mg/dL, or (3) at least one oral glucose tolerance test between 140 and 199 mg/dL, or (4) at least one outpatient International Classification of Diseases, Ninth Revision (ICD-9) code of 790.2, 790.29, 790.21, or 790.22 [5,6]. These laboratory and diagnoses criteria qualified for pre-diabetes only if they were not superseded by the criteria used to meet the definition of diabetes (see below).

Diabetes was defined using the methodology developed for Surveillance, Prevention, and Management of Diabetes Mellitus (SUPREME DM), a large multi-site observational diabetes study [7]. The definition was adapted from the ADA definition of diabetes [5]. Briefly, the definition included one inpatient diagnosis of diabetes or any combination of two other events (outpatient diagnosis, dispense of an anti-hyperglycemic medication, HbA1C equal or greater than 6.5%, fasting plasma glucose equal or greater than 126 mg/dL or random plasma glucose equal or greater than 200mg/dL).

### Hypertension

Hypertension was considered present if an individual had at least two outpatient or one inpatient ICD-9 codes of 401-405xxx.

### Bariatric Surgery

Individuals who had undergone bariatric surgery were identified by an algorithm developed by Arterburn et al in 2009, which used the Current Procedural Terminology 4 (CPT-4) codes (43842, 43843, 43846, 43847), and ICD-9 codes (CPT-4 codes 43659, 43621, 43633) [8]. Verification of this strategy resulted in sensitivity of 99.2% and specificity of 99.9% [8]. Since additional bariatric procedure codes have been created since 2009, the above algorithm was adapted by adding the following codes: 43.82, 43.89, 44.31, 44.38, 44.39, 44.68, 44.69, 44.95, 43633, 43644, 43645, 43770, 43775, 43844, 43845, S2082, S2085. The algorithm was used to search EHR records from the years 2009 to 2013 to identify possible cases of bariatric surgery.

### Charlson Index

Presence of comorbid conditions was assessed with a modified Charlson Comorbidity Index [9-11], which used diagnosis codes for 22 health conditions during the two-year period of January 1, 2012 to December 31, 2013 to create a summary score.

### PORTAL Health Survey

A random sample of 675 overweight and obese English or Spanish reading or speaking individuals were selected from each of the seven KP health plans and Denver Health to complete a brief health survey, for a total of 5400 individuals. An equal number of participants were selected from the categories of overweight, obese class 1, and obese class 2 (n=1080 per category). We randomly selected 2160 for those with obese class 3 and greater, as we were concerned that the extremely obese may not choose to complete the survey. The survey took about 10 minutes to complete and included items on general health and well-being, physical activity, eating patterns, sleep patterns, and perceived health care sensitivities surrounding weight status. The survey was mailed to individuals with telephone follow-up for those who did not return the survey. A US $20 incentive was offered to complete the survey.

**Figure 1:**
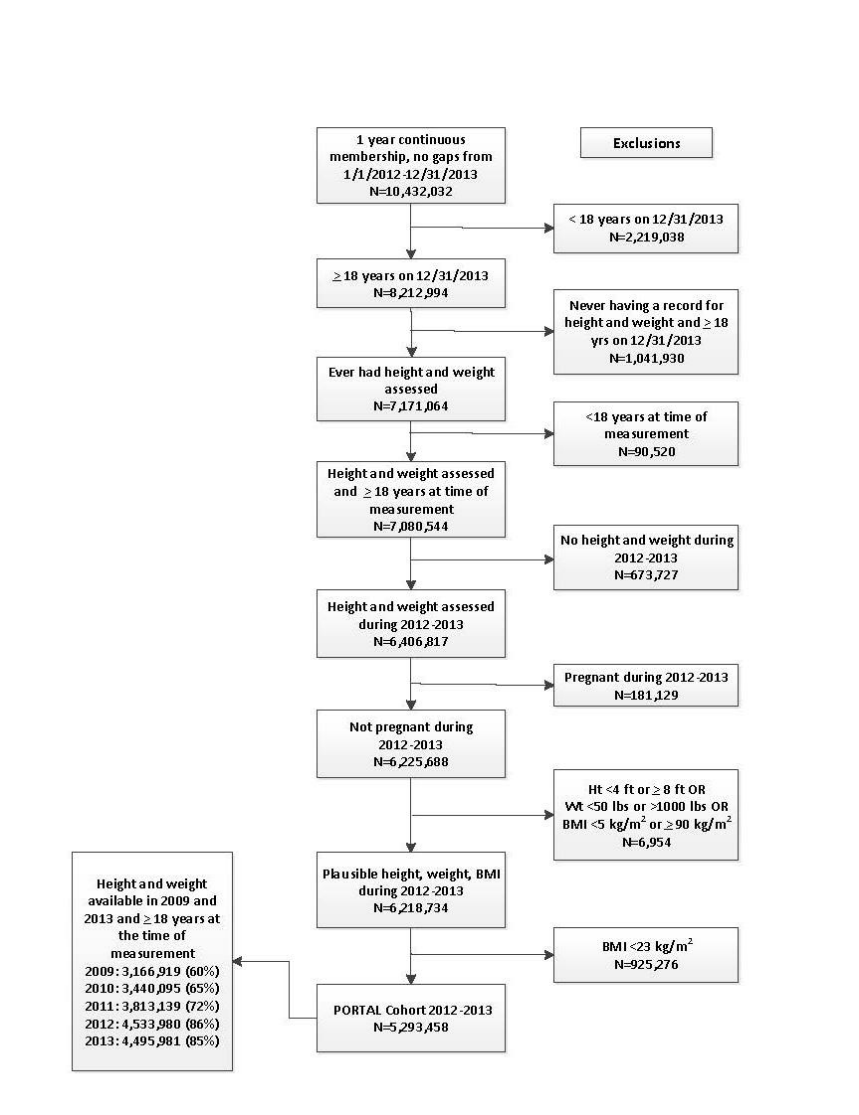
PORTAL overweight and obesity flow chart to construct the cohort.

**Figure 2:**
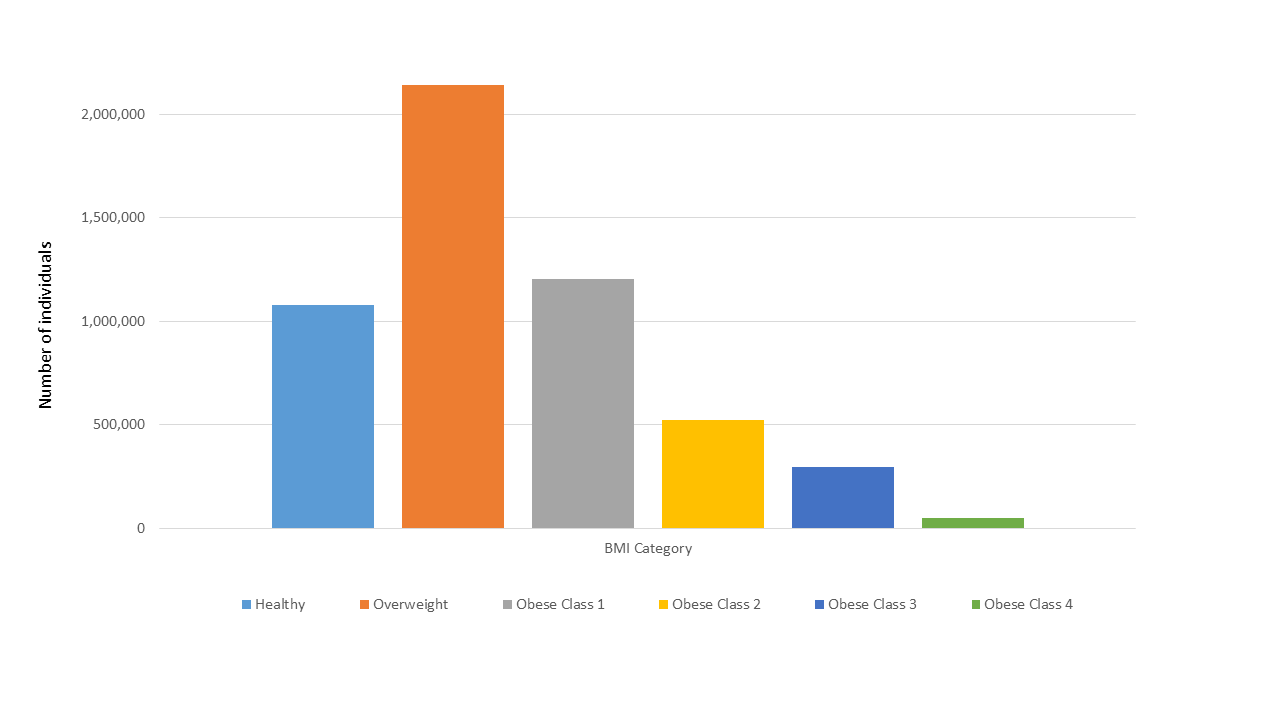
The number of individuals in each BMI category, all PORTAL sites combined.

## Results

### Cohort

The cohort includes over 5 million adults with a BMI >23.0 kg/m^2^. The cohort flow chart with all sites combined is displayed in Figure 1. We identified over 10 million individuals who had continuous membership in 2012 to 2013. After excluding those who were less than 18 years old (n=2,309,558), those who did not have a height and weight recorded (n=1,715,657), who were pregnant during 2012-2013 (n=181,129), and those with implausible height, weight, or BMI measurements (n=6954), a total of 6,218,734 adults remained. We then excluded individuals with a BMI less than 23.0 kg/m^2^ (n=925,276), leaving a cohort of 5,293,458 individuals. A subgroup of the cohort includes a nested cohort of 3,166,919 members who were also enrolled in one of the health plans in 2009 that can be used for future analyses. Although these individuals were members in 2009 and 2013, they may have had different health plan coverage from 2010 to 2012.

Cohort demographics are displayed in Multimedia Appendix 1. Across all network sites 51.95% (2,750,077/5,293,458) are women and 49.02% (2,594,776/5,293,458) are white, 22.89% (1,211,677/5,293,458) are Hispanic, 10.40% (550,608/5,293,458) are Asian, and 10.83% (573,506/5,293,458) are Black. Even though only 1% (75,489/5,293,458) of the cohort is Native Hawaiian/other Pacific Islanders and less than 1% (28,964/5,293,458) is American Indian/Alaskan Natives, they total 75,489 and 28,964 individuals, respectively. The race and ethnicity distribution and neighborhood education and income at each of the ten sites is consistent with the underlying demographics of each region’s population [3-6]. About 2.58% (136,374/5,293,458) of the cohort is insured through a state-subsidized medical insurance plan (eg, Medicaid); another 22.47% (1,189,209/5,293,458) are Medicare recipients. Most individuals (74.81%, 3,959,913/5,293,458) have private insurance, with employer or self-pay options the most prevalent.

Overall, about 85.03% (5,293,458/6,225,688) of non-pregnant individuals over the age of 18 with valid BMI measures obtained in 2012 to 2013 are members of the cohort (Figure 1). The cohort by BMI category, both by the numbers of individuals and prevalence of individuals in each category are shown in Figure 2 and Multimedia Appendix 1. The most common category is overweight, which includes 40.40% (2,138,520/5,293,458) of the individuals in the cohort. The cohort has 52,125 (0.98%, 52,125/5,293,458) persons categorized as obese class 4 (BMI >50 kg/m^2^). The distribution of BMI category is remarkably similar across sites; for example, the prevalence of those in the healthy weight category varied from 17.23% (23,935/138,900) to 21.96% (397,683/1,810,899) and in the obese class 2 category ranged from 8.95% (23,837/266,470) to 11.66% (16,195/138,900) across the 8 sites.

Pre-diabetes varied across sites, with an overall cohort prevalence of 29.49% (1,560,940/5,293,458) and a range from 15.30% (21,248/138,900) to 34.45% (39,171/113,699) across the health plans (Multimedia Appendix 1). Diabetes is prevalent among 14.98% (793,069/5,293,458) of individuals with a range of 12.03% (32,051/266,470) to 20.56% (10,232/49776), and hypertension is prevalent among 34.33% (1,817,436/5,293,458) of individuals with a range of 31.86% (84,886/266,470) to 39.26% (110,560/281,641) in the cohort. Over 25,000 individuals (0.97%, 25,187/5,293,458) were identified as previously having had bariatric surgery.

**Table 1:** Sociodemographic and BMI categories for those who returned the PORTAL health survey (N=2809) compared with those who did not (N=2591).

		Returned survey, n (%)	Did not return survey, n (%)
Sex			
	Female, n=3290	1737 (52.80)	1553 (47.20)
	Male, n=2110	1072 (50.81)	1038 (49.19)
Age category			
	<20 years, n=80	24 (30.00)	56 (70.00)
	20-29 years, n=546	215 (39.38)	331 (60.62)
	30-39 years, n=866	347 (40.07)	519 (59.93)
	40-49 years, n=1115	539 (48.34)	576 (51.66)
	50-59 years, n=1220	680 (55.74)	540 (44.26)
	60-69 years, n=1019	638 (62.61)	381 (37.39)
	70-79 years, n=442	296 (66.97)	146 (33.03)
	>80 years, n=112	70 (62.50)	42 (37.50)
Race/ethnicity			
	White, n=2535	1435 (56.61)	1100 (43.39)
	Hispanic, n=987	420 (42.55)	567 (57.45)
	Asian, n=304	165 (54.28)	139 (45.72)
	Black, n=1144	596 (52.10)	548 (47.90)
	Native Hawaiian/other Pacific Islander, n=301	168 (55.81)	133 (44.19)
	American Indian/Alaskan Native, n=34	20 (58.82)	14 (41.18)
	Other/unknown, n=95	5 (5.26)	90 (94.74)
BMI category			
	Overweight (25.0-29.9 kg/m ^2^), n=1080	577 (53.43)	503 (46.57)
	Obese class 1 (30.0-34.9 kg/m ^2^), n=1080	566 (52.41)	514 (47.59)
	Obese class 2 (35.0-39.9 kg/m^2^), n=1080	550 (50.93)	530 (49.07)
	Obese class 3 (40.0-49.9 kg/m^2^), n=1811	936 (51.68)	875 (48.32)
	Obese class 4 (>50.0 kg/m^2^), n=349	179 (51.29)	170 (48.71)

### Health Survey

From the sample of 5400 individuals, 2809 surveys were completed, 114 were deemed ineligible (ie, no valid address, deceased), 924 persons refused, and 1553 did not respond to mail or telephone attempts, resulting in a 53.14% response of those eligible. Among those who were selected for the survey, women (52.80%, 1737/2809) were slightly more likely to complete the survey than men (50.81%, 1072/2809), and more older individuals returned the survey, for example 62.61% (638/1019) of those age 60 to 69 years completed the survey compared with 39.38% (215/546) of those age 20 to 29 years (Table 1). Completion by race/ethnicity was 59% (20/34) American Indian/Alaskan, 56.61% (1435/2535) White, 55.81% (168/301) Native Hawaiian/Pacific Islanders, 54.28% (165/304) Asians, 52.10% (596/1144) Black, and 42.55% (420/987) Hispanics. There was virtually no difference in response by BMI category, with responses ranging from 50.93% (550/1080) to 53.43% (577/1080) across the five categories, or by self-reported education level.

## Discussion

### Principal Findings

The PORTAL overweight and obesity cohort is large and extends across all regions in the United States. Racial and ethnic diversity, as well as socioeconomic diversity, is large and generally representative of the underlying populations of the health plans’ service regions [12]. The large sample size is particularly useful to support the study of rare exposures or outcomes. Available clinical information is robust and reflects "real world" information that clinicians and health plans use to document health care rather than research quality data collected at pre-specified study intervals. However, prior studies have shown that BMI information collected in the medical record is valid [13]. The cohort can be examined retrospectively and prospectively. For example, exposures identified in 2009 in the sub-cohort can be linked to outcomes identified in 2012 to 2013. The variation across regions, across medical practices, and across different types of health plans with variations in coverage can be examined. A large majority of individuals have access to health insurance (public or private); thus, confounding by health care access is reduced for research focused on health disparities.

The prevalence of individuals across BMI categories and hypertension prevalence was fairly similar across health plans. In contrast, pre-diabetes and diabetes prevalence varied to a greater extent than expected based on hypertension prevalence and BMI status variability. This variability may be due to local differences in testing for pre-diabetes and diabetes, which requires blood work while weight and blood pressure are routinely measured at each visit. The ADA recommends testing for pre-diabetes and diabetes for all adults starting at age 45 years or for those who are overweight and who have additional risk factors, including physical inactivity, hypertension, and being from minority race and ethnicities [5]. However, according to National Health and Nutrition Examination Survey (NHANES) data, only about one-half of those eligible have been tested [14]. Additional research is needed to understand the processes that may explain differences in testing for pre-diabetes and diabetes across sites.

Follow-up of the cohort will be through the clinical information available in EHR. The five year retention is expected to be about 60%, but will vary by health care system. For the 3.1 million individuals who were health plan members in 2009 and 2013, clinical data are available with 5 year follow-up. This information includes repeated measures of height, weight, BMI, prevalent and incident diagnoses from inpatient and outpatient encounters, procedures performed, laboratory test results, pharmaceuticals dispensed, and pathology and radiology results.

PCORnet is created to foster collaborative partnerships across networks and institutions and PORTAL investigators adhere to this principle. The PCORnet CDM (similar to the VDW) has a query function to allow non-PORTAL investigators to inquire about data availability. In general, the information available in the EHR is protected and confidential and remains behind each health plan’s firewall. We welcome external collaborations, particularly collaborations that include establishment of research questions, study design decisions, and analysis and interpretation of the data. Current analyses underway include descriptions of cardiometabolic health among cohort members, incidence of outcomes across BMI categories, and survey results.

### Limitations

In some regions, individuals with low socioeconomic status may be underrepresented, although all health plans except one include individuals covered under state-subsidized insurance, and Denver Health’s mission is to serve those with limited ability to pay for medical services. There is also marginal underrepresentation of those with high incomes. While a large population, the cohort does not include individuals from all 50 states and, therefore, cannot be considered as fully representative of the United States. Because data are collected as part of clinical care, some data elements may not be research quality and are likely to have errors or misclassifications imbedded in them. The classifications of disease status (eg, hypertension, diabetes status) are based on data available in the VDW and have not been chart-reviewed for their validity. However, the quality of diagnosis codes is relatively high in managed care systems and has been validated for many health conditions [15- 17]. The cohort does not include individuals with BMI values less than 23.0 kg/m^2^; therefore we cannot directly compare the cohort to national data sets, such as NHANES.

### Conclusion

The PORTAL overweight and obesity cohort is a rich resource of considerable diversity. It represents the ability of clinical data to be combined across health plans to be available for future epidemiological and comparative effectiveness research.

## Multimedia Appendix 1

Sociodemographic, BMI category, chronic conditions, and health insurance status across the 10 PORTAL obesity cohort sites (N= 5,293,458).

## Abbreviations

ADA: American Diabetes Association
BMI: body mass index
CDRN: Clinical Data Research Network
CDM: Common Data Model
CPT-4: Current Procedural Terminology 4
EHR: Electronic Health Records
HbA1c: glycated hemoglobin
ICD-9: International Classification of Diseases, Ninth Revision
IRB: institutional review board
KPSC: Kaiser Permanente Southern California
NHANES: National Health and Nutrition Examination Survey
PCORI: Patient-Centered Outcomes Research Institute
PCORnet: Patient-Centered Clinical Research Network
PORTAL: Patient Outcomes Research to Advance Learning
VDW: Virtual Data Warehouse
WHO: World Health Organization
