# Supplementary material for: The Patient Outcomes Research To Advance Learning (PORTAL) Network Adult Overweight and Obesity Cohort: Development and Description
Source: JMIR Res Protoc. 2016 Jun 15;5(2):e87. doi: 10.2196/resprot.5589 (PMC4927804; doi:10.2196/resprot.5589)
Supplement: Supplementary file 1 [file resprot_v5i2e87_app1.pdf]

Appendix Table. Sociodemographic, BMI category, chronic conditions, and health insurance status across the 10 PORTAL obesity cohort sites (N= 5,293,458).

		Site[a], %										Total
Variable		1	2	3	4	5	6	7	8	9	10	
Sex												
	Female	51	53	51	52	54	54	48	63	56	53	52
	Male	49	46	49	48	46	46	52	37	45	46	48
Race/ ethnicity												
	White	38	67	53	81	41	32	27	26	78	79	49
	Hispanic	37	12	19	6	4	9	6	53	5	1	23
	Asian	8	2	15	4	5	7	34	3	8	4	10
	Black	10	4	8	3	47	40	1	18	5	9	10
	Native Hawaiian/other Pacific Islander	1	0	1	1	0	0	27	N/A[b]	1	0	1
	American Indian /Alaskan Native	0	1	<1	1	0	<1	<1	<1	2	1	<1
	Unknown/other	5	10	4	4	3	11	5	0	3	4	5

Age, mean (SD)		50 (17)	52 (17)	51 (18)	51 (17)	48 (17)	50 (17)	51 (17)	45 (16)	53 (17)	50 (18)	50 (17)
BMI category												
	Healthy[c]	20	21	22	18	17	18	22	18	20	19	20
	Overweight[c]	41	42	41	38	38	38	39	38	39	39	40
	Obese class 1[c]	24	22	22	24	24	24	21	24	22	23	23
	Obese class 2[c]	10	9	9	12	12	11	10	11	11	11	10
	Obese class 3[c]	5	5	5	8	7	7	6	7	6	6	6
	Obese class 4[c]	1	1	1	2	1	2	1	2	1	1	1
Charlson comorbidity score, mean (SD)		0.5 (0.9)	0.8 (1.3)	0.8 (1.3)	0.7 (1.2)	0.6 (1.1)	0.7 (1.2)	0.8 (1.3)	0.7 (1.2)	0.8 (1.3)	0.7 (1.2)	0.7 (1.1)
Hypertension		34	32	35	33	36	39	36	36	35	33	36
Pre-diabetes		32	23	31	30	15	32	35	22	17	17	30
Diabetes		16	12	15	14	14	17	18	21	14	12	15

Bariatric surgery		1	1	0	0	0	0	0	0	0	0	1
Neighborhood median family income category, US $												
	<15,000	7	5	5	7	8[d]	4	5	14	5	5	6
	15,000 to $34,999	16	12	13	15	17	9	12	25	12	12	14
	35,000 to 49,999	13	11	10	13	13	9	11	15	11	11	11
	50,000 to 74,999	18	19	17	21	20	15	19	18	19	19	18
	75,000 to 99,999	14	16	15	16	15	14	17	11	16	17	15
	100,000 to 149,999	18	20	20	18	16	22	21	10	21	21	19
	≥150,000	14	15	19	11	11	26	14	7	16	14	16

Neighborhood education												
	<9th grade	11	4	7	4	5	5	5	12	3	3	8
	9th-12th grade	9	6	7	6	8	6	5	12	5	4	8
	High school graduate	22	22	21	24	26	22	30	25	22	25	22
	Some college	23	22	22	27	21	20	22	19	25	22	23
	Associate degree	8	8	8	9	7	6	10	5	9	9	8
	Bachelor degree	18	25	22	20	22	23	19	17	23	24	21
	Graduate or professional degree	8	12	11	10	12	17	8	9	12	11	10
Health insurance												
	State subsidized	2	3	2	2	1	0	6	27	0	12	3

	Medic are	20	28	25	26	16	21	23	7	29	17	23
	Other	78	69	73	73	84	79	71	66	71	71	75

[a]Patient populations of each site; 1: 1,993,104, 2: 266,470, 3:1,810,899, 4: 279,302, 5: 138,900, 6: 281,641, 7: 113,699, 8:49,776, 9: 236,193, 10:123,474.

[b]This health plan does not separate Native Hawaiians/other Pacific Islanders from the Asian category.

[c]Healthy (23.0 – 24.9 kg/m^2), overweight (25.0-29.9 kg/m^2), obese class 1 (30.0-34.9 kg/m^2), obese class 2 (35.0-39.9 kg/m^2), obese class 3 (40.0-99.9 kg/m^2), obese class 4 ($\geq$50.0 kg/m^2).

[d]This health plan was missing 53% of census data used to estimate neighborhood family income and neighborhood education level. Imputed values replacing the missing with the mean probability values resulted in similar prevalences as the unimputed values. Unimputed values are presented.
